# Therapeutic potential of mitochondrial transplantation in modulating immune responses post-cardiac arrest: a narrative review

**DOI:** 10.1186/s12967-024-05003-2

**Published:** 2024-03-03

**Authors:** Tomoaki Aoki, Yusuke Endo, Eriko Nakamura, Cyrus E. Kuschner, Jacob Kazmi, Parmeshar Singh, Tai Yin, Lance B. Becker, Kei Hayashida

**Affiliations:** 1 Laboratory for Critical Care Physiology, Feinstein Institutes for Medical Research, Northwell Health System, Manhasset, NY USA; 2 Department of Emergency Medicine, Donald and Barbara Zucker School of Medicine at Hofstra/Northwell, Hempstead, NY USA

**Keywords:** Heart arrest, Cardiopulmonary resuscitation, Ischemia, Reperfusion injury, Inflammation, Immune response, Mitochondrial transplantation

## Abstract

**Background:**

Mitochondrial transplantation (MTx) has emerged as a novel therapeutic strategy, particularly effective in diseases characterized by mitochondrial dysfunction. This review synthesizes current knowledge on MTx, focusing on its role in modulating immune responses and explores its potential in treating post-cardiac arrest syndrome (PCAS).

**Methods:**

We conducted a comprehensive narrative review of animal and human studies that have investigated the effects of MTx in the context of immunomodulation. This included a review of the immune responses following critical condition such as ischemia reperfusion injury, the impact of MTx on these responses, and the therapeutic potential of MTx in various conditions.

**Results:**

Recent studies indicate that MTx can modulate complex immune responses and reduce ischemia–reperfusion injury post-CA, suggesting MTx as a novel, potentially more effective approach. The review highlights the role of MTx in immune modulation, its potential synergistic effects with existing treatments such as therapeutic hypothermia, and the need for further research to optimize its application in PCAS. The safety and efficacy of autologous versus allogeneic MTx, particularly in the context of immune reactions, are critical areas for future investigation.

**Conclusion:**

MTx represents a promising frontier in the treatment of PCAS, offering a novel approach to modulate immune responses and restore cellular energetics. Future research should focus on long-term effects, combination therapies, and personalized medicine approaches to fully harness the potential of MTx in improving patient outcomes in PCAS.

## Background

Mitochondrial transplantation (MTx) is an emerging therapeutic strategy demonstrating profound efficacy in a spectrum of diseases, primarily those rooted in mitochondrial dysfunction [1, 2]. This cutting-edge technique involves the transfer of functional mitochondria from healthy donor to compromised recipient tissues, effectively restoring mitochondrial function, and ameliorating disease progression [3–6]. Moreover, recent discoveries regarding the natural, extracellular transfer of mitochondria between cells open new therapeutic vistas [7]. With its applicability extending to both genetic and acquired disorders, MTx represents a transformative approach for addressing the mitochondrial insufficiencies central to disease pathogenesis [8]. This modality holds substantial promise for future clinical translation, with the potential to significantly enhance patient outcomes.

Cardiac arrest (CA) poses a multifaceted clinical challenge, characterized by systemic ischemia–reperfusion (IR) injury, which triggers a cascade of intricate physiological alterations to vital organs [9–12]. Among these multifaceted changes, the activation of immune responses is increasingly recognized as a pivotal determinant [13–15]. In addition, accumulating evidence implicates IR-induced mitochondrial dysfunction as a key pathophysiological mechanism during the post-CA phase [16–18]. This mitochondrial dysfunction might be instrumental in driving the observed aberrations in immune cell function following CA. We have recently demonstrated that MTx improves outcomes post-CA in a rodent model [19]. Of note, emerging data suggest that MTx possibly serves a dual purpose: it not only replenishes dysfunctional mitochondria but also modulates immune responses. Thus, deep insights into the interplay between MTx and immune response during IR hold the potential to revolutionize therapeutic strategies for IR injury. In this context, we offer a comprehensive narrative review aimed at elucidating the role of MTx in modulating immune responses post-CA.

## Animal studies elucidating immune responses following cardiac arrest and resuscitation

While the immune system is essential for infection control and homeostasis, CA resuscitation disrupts immunological functions in a complex manner. It triggers a systemic inflammatory response, characterized by a surge in inflammatory mediators and cytokines. While this response is integral for tissue repair and cellular debris clearance, its dysregulation can exacerbate organ damage and lead to post-CA syndrome (PCAS). Numerous studies corroborate that CA often results in immune alterations, evidenced by increased neutrophil and monocyte activity, compromised lymphocyte proliferation, and altered cytokine profiles [14, 20, 21]. These disruptions further exaggerate IR injury after CA, emphasizing the urgent need for innovative strategies to mitigate immune-related complications and improve post-CA outcomes.

The intricate relationship between CA and immune responses in the context of brain injury has been elucidated [22]. Upon injury, microglia, the brain's resident immune cells, swiftly activate, releasing substances with dual cytoprotective and cytotoxic properties, including pro-inflammatory cytokines such as interleukin (IL)-1ꞵ, IL-6, IL-10, and interferon (IFN)-γ [23–25]. This activation triggers the infiltration of peripheral immune cells, such as neutrophils, macrophages, and T lymphocytes, exacerbating neuronal damage [25–27]. Astrocytes, supportive brain cells, also respond to hypoxic stress by releasing both neuroprotective and pro-inflammatory cytokines, potentially leading to delayed neuronal death [28, 29]. Notably, neutrophils and T lymphocytes, including CD4^+^ and CD8^+^ T cells, participate in cerebral IR injury, promoting inflammation, brain infarction, and neurological deficits [30–32]. While these immune responses initially aim to safeguard the brain, they can paradoxically contribute to ongoing neuronal injury and delayed damage [33].

In the context of global cerebral ischemia caused by CA resuscitation, the traditionally immune-privileged brain experiences rapid infiltration of pro-inflammatory T cells [34]. These infiltrating cells are predominantly CD4^+^ T lymphocytes, which not only attain an activated state but sustain their presence in the cerebral parenchyma for an extended period of time, up to 72 h post resuscitation [35, 36]. In studies of experimental ischemic stroke, the timing of immune cell entry into the brain has been well-documented. Specifically, neutrophils are the first to arrive, presenting rapidly within several hours after ischemic strokes, followed by lymphocytes which appear 12 to 24 h after neuronal ischemic injury [35–38]. This lymphocytic infiltration is concomitant with a marked exacerbation of neuronal cell death in the hippocampal CA1 region, a phenomenon that may be mediated by the secretion of pro-inflammatory cytokines such as TNF-α and IFN-γ [37, 39]. Consequently, these observations suggest that modulating T cell infiltration and the ensuing immune cascade in the aftermath of CA could serve as a novel therapeutic avenue [32].

In a study using a swine CA model, increased pro-inflammatory cytokines, such as IL-6 and TNF-α, were observed in renal tissue post-CA. T lymphocytes, specifically CD4^+^ and CD8^+^ T cells, were shown to infiltrate renal tissue, contributing to post-CA renal injury. Severity of renal injury correlated with T lymphocyte infiltration and cytokine production, with prolonged resuscitation leading to more pronounced damage [40]. Further, regulatory T cells (Treg), responsible for immune regulation and inflammation suppression, decreased in renal tissue post-CA, disrupting a balanced immune response. These observations suggest that activation of T lymphocytes, altered Treg function, and immune disruption can contribute to post-CA renal damage, offering potential therapeutic targets for mitigating organ injury after CA resuscitation.

Furgeson et al. demonstrated that peripheral mitochondrial bioenergetic profiles correlate with degree of injury post CA. Using a piglet model of pediatric CA, these authors studied the association of peripheral platelets on brain injury after CA resuscitation [41]. They revealed that CA leads to significant enhancements in platelet mitochondrial bioenergetics, primarily through increased respiration via complex II of the electron transport system [41]. Platelet mitochondrial respiratory capacity and efficiency also rise post-CA. This suggests that platelet mitochondrial respiration, measured from peripheral blood, may serve as a non-invasive biomarker for assessing post-CA cerebral bioenergetic dysfunction. Given mitochondrial damage-associated molecular patterns (DAMPs) ability to activate platelets and prompt inflammation [42, 43], CA may induce platelet mitochondrial biogenesis, but increased functional respiration appears to result from post-translational modifications and other mechanisms.

Another study showed that CA activates the hypothalamic–pituitary–adrenal (HPA) axis, a vital stress response pathway, resulting in the release of stress hormones, including cortisol, which modulate immune responses [44]. Prolonged CA resuscitation duration directly correlated with increased severity of immunosuppression and immune dysfunction. A mechanistic link between post-CA immunosuppression and the HPA axis was supported by evidence that glucocorticoid treatment exacerbates immunosuppression. Conversely, RU486 (Mifepristone), a synthetic steroid that acts as a glucocorticoid receptor antagonist and a progesterone receptor antagonist, was found to mitigate lymphopenia and atrophy, improving CA outcomes.

A mouse model reveals that asphyxia CA severely impairs lymphopoiesis, reducing systemic lymphocyte production in the bone marrow and thymus, which are critical for lymphocyte development [45]. This impairment persists after resuscitation and, importantly, this study uncovered potential mechanisms behind this impairment. They found that CA increased reactive oxygen species (ROS) and DNA damage in the bone marrow and thymus, known to negatively impact lymphocyte development. Additionally, IL-7, crucial for lymphocyte development, significantly decreased in these organs post-CA, potentially contributing to the observed lymphopoiesis impairment.

The above-mentioned animal studies demonstrate that the multifaceted interplay between CA resuscitation and immune responses presents a complex landscape that is crucial for understanding PCAS and organ damage. The evidence suggests that CA resuscitation triggers a systemic inflammatory response that, while essential for tissue repair and cellular debris clearance, can lead to immune dysregulation and exacerbate IR injuries. This is particularly evident in the brain and systemic circulation, where immune cells such as T lymphocytes and neutrophils, as well as inflammatory cytokines, play pivotal roles. The activation of the HPA axis and its modulation by glucocorticoids, as well as peripheral platelet mitochondrial bioenergetics and lymphopoiesis impairment, further add complexity to the immune landscape post-CA. These findings collectively underscore the urgent need for innovative strategies aimed at modulating immune responses to improve outcomes in post-CA care.

## Prognostic assessment of post-cardiac arrest outcomes through targeted evaluation of immune response mechanisms

In a study of out-of-hospital CA (OHCA) survivors, Qi et al. studied presepsin as a prognostic biomarker for early immune changes [46]. They discovered that presepsin levels surged after return of spontaneous circulation (ROSC), with higher levels associated with unfavorable neurological outcomes and mortality. Both presepsin and procalcitonin exhibited distinct patterns in the first three days post-ROSC among patients with different outcomes. This combination of biomarkers was identified as independent predictors of 28-day survival and favorable neurological outcomes. Additionally, the study examined monocyte markers, CD14 and HLA-DR, revealing that CD14 expression increased on day 1 but decreased on day 3, while HLA-DR expression consistently decreased on both days 1 and 3. These changes correlated with TLR-4 expression. Together these findings support a transient hyperactivity and subsequent dysfunction of innate immune functions.

In a study examining the relationship between immune responses and survival in patients with CA, investigators focused on CD73-expressing lymphocytes, a heterogenous population of adenosine-producing CD4^+^ and CD8^+^ T cells, naïve and memory B cells, and follicular dendritic cells [47–50]. Their investigation aimed to determine if the levels of these lymphocytes could serve as a survival predictor, revealing that survivors had significantly higher circulating CD73-expressing lymphocyte levels compared to non-survivors [47]. In addition, the number of these lymphocytes correlated with favorable neurological outcomes in survivors. Higher CD73 expression on lymphocytes led to increased adenosine production, which possesses anti-inflammatory and tissue-protective properties [47], suggesting that elevated CD73-expressing lymphocyte levels are associated with improved survival and neurological function in patients with CA. The role of CD73 as a marker of the body’s ability to quell immune responses is further supported by both animal and human studies that employ antibody-mediated experimental blockade to reactivate adaptive immune responses in cancer [50, 51].

A recent study of serum samples collected from Swedish patients enrolled in the targeted temperature management (TTM) trial has identified additional, potential biomarkers through proteomic analysis [52]. Comparing patients with good and poor neurological outcome 6 months following CA, the study proposed the utility of proteins known to be implicated in immune programs, including kallistatin and angiotensinogen, in neuroprognostication and treatment monitoring. While these markers have not been validated or investigated in larger cohorts, further studies may help to advance them into clinical practice.

## Human studies aiming to regulate excessive immune responses following cardiac arrest resuscitation

Clinical studies highlight the potential of Cyclosporine A (CsA) and tocilizumab in modulating immune responses post-CA, offering new directions for PCAS treatment strategies [53]. CsA, when administered at the commencement of cardiopulmonary resuscitation (CPR), has demonstrated short-term benefits by reducing multi-organ dysfunction, supported by the CYRUS trial's findings on mitigating post-CA immune dysfunction without affecting survival in non-shockable OHCA cases [54, 55]. The IMICA trial further revealed tocilizumab's effectiveness in lowering inflammatory markers and cardiac injury indicators, although its impact on long-term outcomes and neurocognitive functions remains unclear [56, 57]. These findings suggest a promising role for these interventions in improving short-term outcomes, yet the long-term effects require further investigation.

Glucocorticoids, particularly hydrocortisone, have shown promise in various trials targeting CA patients, enhancing ROSC rates, reducing organ dysfunction, and improving survival [58, 59]. These steroids appear to lower inflammation by decreasing pro-inflammatory cytokines [60, 61]. Further, a prospective observational single-center study focused the immune responses and glucocorticoid receptor expression in patients with CA [62]. The study revealed that CA survivors exhibit lower glucocorticoid receptor levels in leukocytes, especially those with prolonged CA and adverse neurological outcomes, linking receptor downregulation with increased inflammation [62]. This suggests that early post-CA corticosteroid therapy might help regulate immune responses and improve patient outcomes by normalizing glucocorticoid receptor activity.

## Therapeutic hypothermia and immune response after cardiac arrest resuscitation

TTM, inclusive of therapeutic hypothermia (TH), is the primary neuroprotective approach post-CA, with potential synergies with mitochondrial therapies to enhance recovery. While neuroprotective effects of TH are established, optimizing its application, including temperature and timing, is crucial to maximize benefits and minimize risks. Early and sustained TH is essential for improving outcomes, highlighting the need for strategic application [63].

Recent studies have explored the impact of induced hypothermia on post-CA immune modulation. Ultrafast hypothermia, applied immediately after CA, has been shown to reduce pro-inflammatory cytokines like IL-6 and TNF-α, suggesting its potential in mitigating PCAS-related inflammation [64]. However, the immunomodulatory effects of hypothermia are complex, with some studies indicating transient cytokine increases without significant changes in immune markers, such as HLA-DR [65, 66]. These findings point to the nuanced role of hypothermia in immune responses post-CA, underscoring the necessity for further research to clarify its therapeutic implications.

## Therapeutic potential of mitochondrial transplantation for immune response modulation in various conditions

**Fig. 1:**
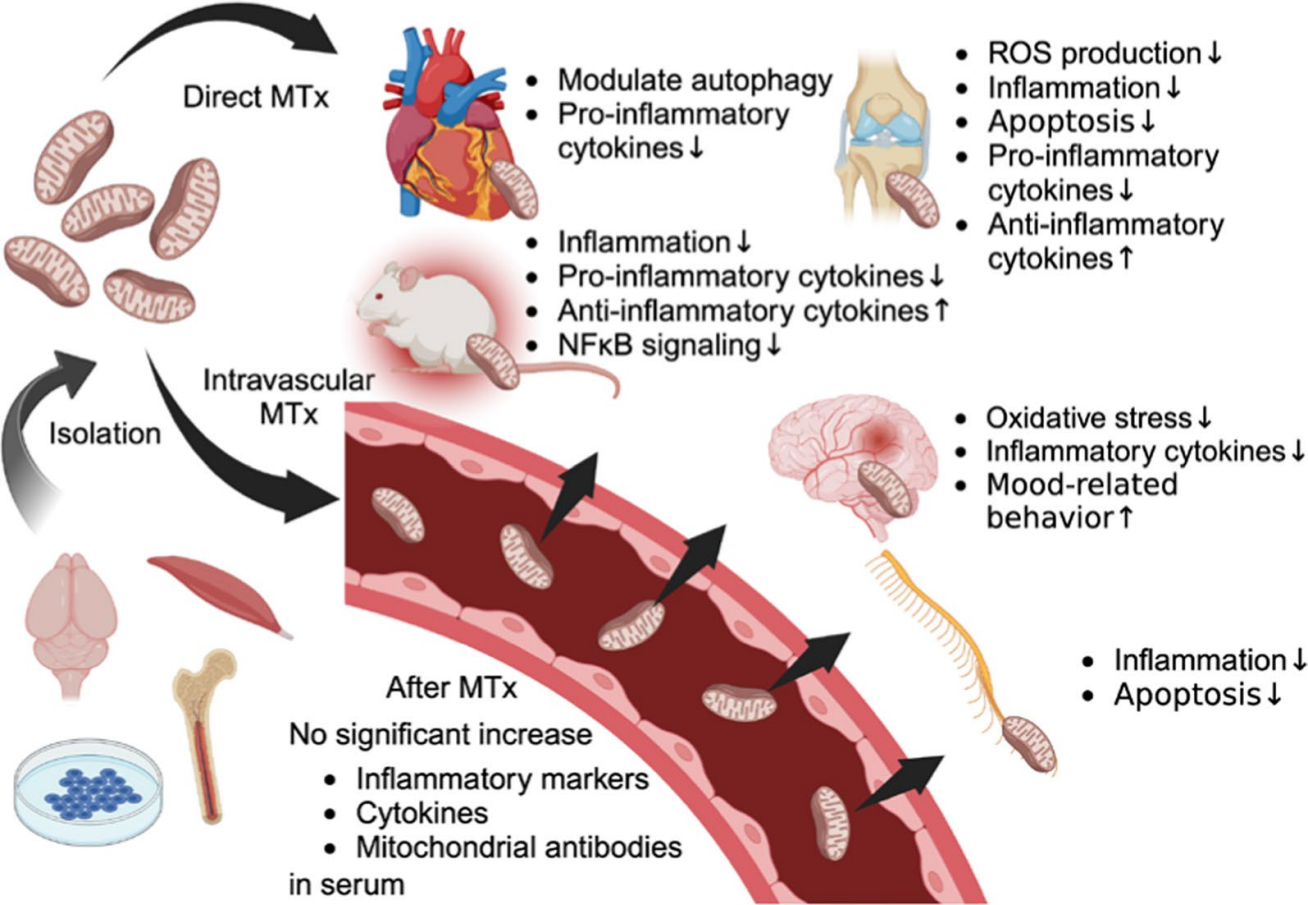
Therapeutic potential of mitochondrial transplantation in modulating immune responses. Created with BioRender.com

As summarized in Table 1 and depicted in Fig. 1, therapeutic potentials of MTx have been shown in various conditions. Based on the observation that cells can assimilate mitochondria from their surroundings, MTx has emerged as a strategy with the potential to modulate the immune response in various pathological conditions [67]. In cardiovascular disease the role of extracellular mitochondrial components has been highlighted, with their potential to regulate immune responses and promote cell survival, suggesting MTx as a new avenue for therapeutic intervention [68]. Furthermore, in several disease models, MTx has been shown to regulate the inflammatory response effectively. Studies using rodent sepsis models, for instance, demonstrated that MTx improved bacterial clearance and survival outcome, indicating immunomodulatory effects [69, 70]. MTx was associated with decreased bacterial burden, systemic inflammation, complement and coagulation cascades, and organ injury [69]. MTx also exhibits a bimodal immunomodulatory effect; it reduces immune cell apoptosis and immune paralysis, enhancing bacterial clearance and survival, while also attenuating hyperinflammatory responses early in sepsis [70]. This dual action suggests that MTx can regulate immune function by shifting cell metabolism, potentially offering a single therapeutic strategy for the dynamic immune changes in sepsis, from hyperinflammation to immune paralysis. A study on 22–24 month old rats with myocardial IR injury revealed that combining Coenzyme Q10 (ubiquinone; a lipid-soluble molecule functioning as an electron carrier in the mitochondrial electron transport chain) with MTx improved both short-term and long-term outcomes, with enhanced autophagic flux and reduced inflammatory response following the injury [71]. In another study, allogeneic MTx in rats with traumatic spinal cord injury showed promising therapeutic effects. Labeled mitochondria were present in the injured spinal cord for up to 28 days post transplantation. Rats receiving this treatment showed improved locomotor and sensory functions, reduced expression of dynamin-related protein 1, less demyelination, and alleviated cellular apoptosis and inflammation in the injured cord. These results suggest that early-stage allogeneic MTx in spinal cord injury can mitigate mitochondrial damage, neuroapoptosis, neuroinflammation, and oxidative stress, thereby enhancing functional recovery [72]. One study using mice lung IR injury model demonstrated that donor mitochondria either directly injected into the pulmonary artery or via nebulization attenuated neutrophil infiltration, interstitial edema, and apoptosis in lungs [73]. However, the study reported no significant differences in cytokines and chemokines levels in bronchoalveolar lavage fluid at 24 h post reperfusion between the groups that received MTx and those that did not. This suggests that the impact of MTx may be more pronounced in physical tissue changes rather than in the modulation of these specific inflammatory markers in this particular model at the measured time point.

**Table 1:** Summary of the mitochondrial transplantation studies described in the present review

Species	Disease models	Targeted organs	Mitochondrial sources	Transplantation methods and timings	Immune response-related outcomes	Refs.
New Zealand white rabbits (male)	Focal ischemia	Heart	Autograft: Pectoralis major muscle	Direct injection 8 times, 1 min before reperfusion	No significant increase in serum inflammatory markers, No anti-mitochondrial antibodies in serum	[80]
Yorkshire pigs (female)	Focal ischemia	Heart	Autograft: Pectoralis major muscle	Direct injection 8 times, 1 min before reperfusion	No significant increase in serum cytokines	[81]
Wistar rats (male)	Focal ischemia	Heart	Allograft: Muscle	Direct injection, upon reperfusion	Modulated autophagic activity, decreased pro-inflammatory cytokines	[71]
1) Wistar rats 2) patient-derived hLCLs	1) Poly I:C MIA 2) Schizophrenia	1) Brain 2) Cell	1) Allograft: Brain 2) hLCLs	1) IC 2) Co-culture	MTx to Poly I:C MIA showed improved immune balance MTx to healthy hLCLs exhibited immune imbalance, and stress-related chemokine release	[79]
ICR mice (male)	Depression	Brain	Allograft: Hippocampus	IV, upon or 6 h after LPS injection	Decreased oxidative stress and inflammatory cytokine levels, improved mood-related behavior	[75]
SD rats	Focal ischemia	Spinal cord	Allograft: Soleus muscle	Direct injection 2 times, Following SCI	Alleviated cellular apoptosis and inflammation responses	[72]
SD rats (male)	Tendinopathy	Tendon	Allograft: L6 rat myoblast cell lines	Direct injection, 2 weeks after collagenase injection	Reduced inflammation, and apoptosis; Decreased pro-inflammatory cytokines, increased anti-inflammatory cytokines	[76]
SD rats (male)	Osteoarthritis	Knee joint	BMSCs from tibia and femur	Direct injection into joint cavity, Once a week for 6 weeks	Increased the production of ATP, inhibited apoptosis, and decreased the production of ROS	[77]
C57BL/6 mice (male)	Sepsis	Whole body	Allograft: Pectoralis major muscle	IV (tail vein), 120 min after CLP	Reduced inflammation, enhanced bacterial clearance, decreased pro-inflammatory cytokines, and increased anti-inflammatory cytokines	[69]
BALB/C mice (male)	Sepsis	Whole body	UC-MSCs	IV, upon or 30 min after LPS injection	Inhibited the LPS-induced production of pro-inflammatory cytokines/ chemokines, Inhibited the NFκB signaling pathway	[78]

*hLCLs* human lymphoblastoid cell lines, *Poly I:C MIA* polyinosinic:polycytidylic acid maternal immune activation, *IC* intracerebral injection, *MTx* mitochondrial transplantation, *ICV* intracerebroventricular injection, *IV* intravenous injection, *LPS* lipopolysaccharide, *SD* Sprague–Dawley, *SCI* spinal cord injury, *BMSCs* bone marrow mesenchymal stem cells, *ATP* adenosine triphosphate, *ROS* reactive oxygen species, *CLP* Cecal ligation and puncture, *UC-MSCs* umbilical cord-derived mesenchymal stem cells, *NFκB* nuclear factor kappa-light-chain-enhancer of activated B cells

The anti-inflammatory and regenerative capabilities of MTx have also been evidenced in a range of non-critical ill conditions. In chronic neurodegenerative diseases, MTx has been proposed as a means to counteract the chronic inflammation that contributes to neuronal damage, with the potential to alter the disease course in conditions such as Alzheimer's and Parkinson's disease (PD) [74]. In a mouse model, transplantation of healthy mitochondria into the hippocampus attenuated lipopolysaccharide (LPS)-induced depression-like behaviors by enhancing mitochondrial function, reducing oxidative stress and inflammatory cytokines, thereby suggesting potential therapeutic efficacy for depressive disorders linked to inflammation and mitochondrial dysfunction [75]. In tendinopathy, transplanted mitochondria have been shown to increase tenocyte integrity markers, reduce TNF-α to control levels and dramatically reduce IL-1β and IL-6 levels, thereby facilitating tissue regeneration [76]. Similarly, in osteoarthritis, mitochondria derived from bone marrow mesenchymal stem cells have been found to enhance chondrocyte function and stimulate mitochondrial biogenesis, contributing to joint health [77]. Human MSC-derived mitochondria have shown promise in attenuating LPS-induced inflammatory responses by inhibiting the nuclear factor kappa-light-chain-enhancer of activated B cells (NFκB) signaling pathway [78]. Further, a link between mitochondrial health in neurodevelopment and brain functioning in adulthood was established in a maternal immune activation model of schizophrenia in rats [79]. Intracerebral injection of allogenic, healthy mitochondria into the medial prefrontal cortex of adolescent rats revealed disparate initial changes in mitochondrial function and inflammatory responses between schizophrenic and control rats. These were associated with opposite effects in proteome alteration, monoamine turnover, neuronal sprouting and behavior in adulthood, dependent on disease state, supporting the therapeutic potential of MTx during adolescence in neurodevelopmental disorders.

As for potential adverse immune reactions due to MTx, studies have shown that autologous MTx, as applied in a rabbit model of ischemic cardiomyopathy and a porcine model of ischemia/reperfusion, did not elicit significant immune responses or elevate serum cytokine levels, suggesting its relative safety and low immunogenicity [80, 81]. Moreover, no discernible instances of direct or indirect, acute or chronic alloreactivity, allorecognition or DAMPs were observed following single or serial injections of either syngeneic or allogeneic mitochondria in mice [82]. Similarly, both autologous and allogenic MTx were executed without adverse effects in diverse models of organ ischemia reperfusion injury [4, 73, 83–87]. However, the safety and efficacy of autologous versus allogeneic MTx in humans, particularly in the context of immune responses, represent crucial domains necessitating future exploration.

**Fig. 2:**
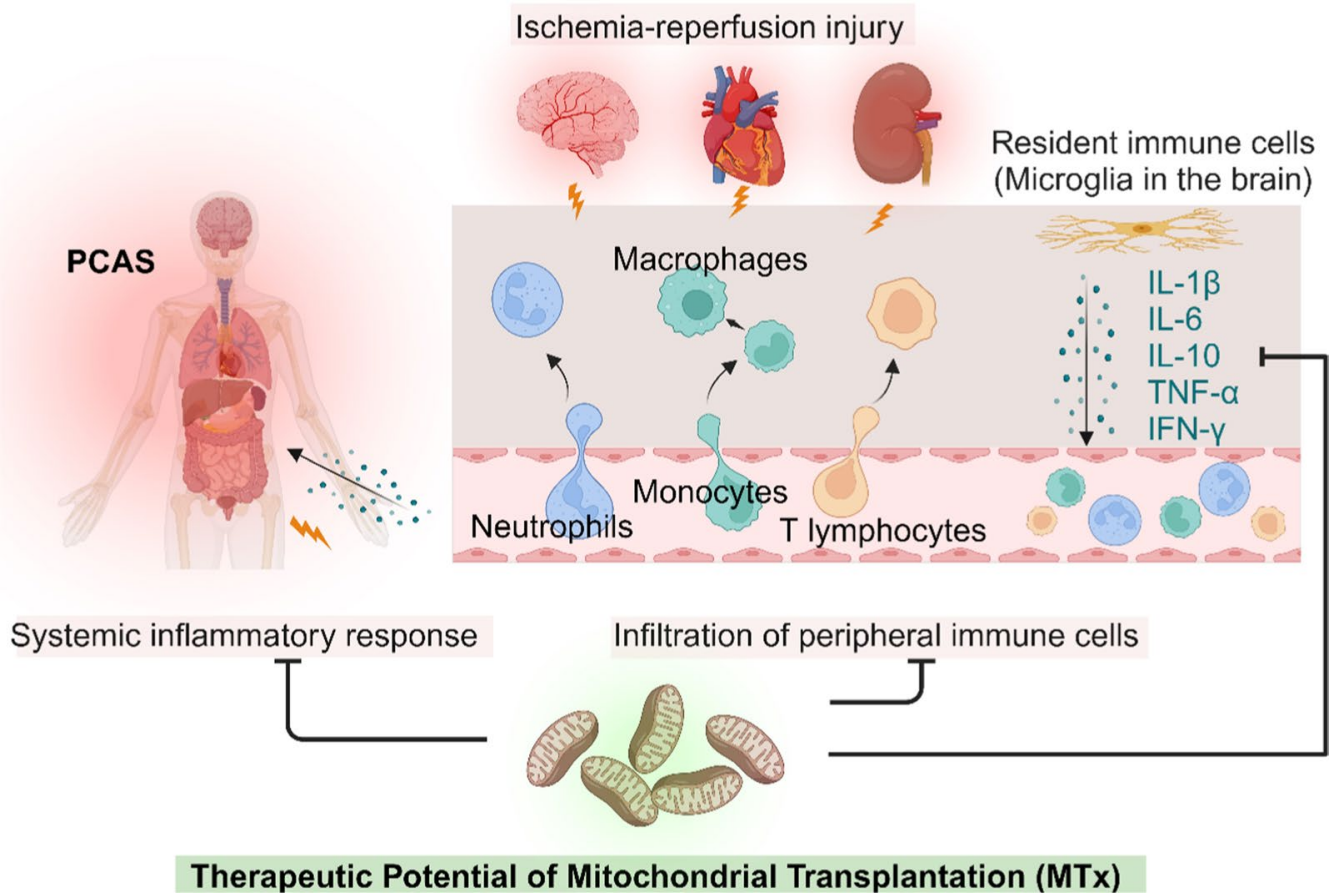
Therapeutic potential of mitochondrial transplantation in modulating immune responses post CA. Created with BioRender.com

Taken together, MTx represents a frontier in the modulation of the immune response, offering a novel approach to the treatment of a wide array of diseases characterized by inflammatory alterations. The ability to restore cellular energetics as well as modulate the immune system provides a unique therapeutic avenue, with the potential to revolutionize the management of critical illnesses, including PCAS. The dual efficacy of MTx in enhancing cellular energy and regulating immune responses after CA is illustrated in Fig. 2.

## Future directions in mitochondrial transplantation for post-cardiac arrest syndrome

The promising results from preclinical and early clinical studies on MTx necessitate the expansion of further studies specifically investigating PCAS. Moreover, a deeper understanding of the mechanisms by which MTx modulates immune responses in the context of PCAS is essential. Future research should aim to elucidate the intricate interactions between transplanted mitochondria and various immune cells, and how these interactions influence the overall immune response post-CA. This includes studying the role of mitochondrial-derived DAMPs and their impact on inflammation and tissue repair. The presence and the degradation of circulating cell-free intact mitochondria originating from several cell types in plasma were demonstrated in both physiological and pathological conditions, which were presumed to be capable of activating immune cells and modulating an inflammatory response [88, 89]. Intravenously injected exogenous mitochondria were also demonstrated to distribute in various tissues including brain, liver, kidney, muscle and heart in experimental PD model mice, which prevented PD progress through increasing the activity of electron transport chain, decreasing ROS level, and preventing cell apoptosis and necrosis [90]. This suggests that MTx would benefit on the multi-organ injury in PCAS. Additionally, exploring the potential of MTx in modulating the balance between pro-inflammatory and anti-inflammatory responses could lead to more effective strategies for managing the complex immune dysregulation observed in PCAS.

## Conclusions

The burgeoning field of MTx presents a novel and promising therapeutic avenue for addressing the complex pathophysiology of PCAS. By focusing on long-term effects, combination therapies, and a deeper understanding of the immunomodulatory mechanisms of MTx, this innovative treatment could revolutionize the management of PCAS. The ability of MTx to restore cellular energetics and modulate immune responses offers a unique and multifaceted approach to tackling the challenges of PCAS, potentially leading to enhanced survival rates, improved neurological outcomes, and better quality of life for patients. Further exploration to elucidate detailed mechanism and relationship between MTx and immune responses post-CA, which might pave the way to facilitate clinical implementation of MTx for patients suffering from CA pathophysiology, are warranted.

## Data Availability

Not applicable.

## Abbreviations

ATP: Adenosine triphosphate
CA: Cardiac arrest
CPR: Cardiopulmonary resuscitation
CsA: Cyclosporine A
DAMPs: Damage-associated molecular patterns
HPA: Hypothalamic–pituitary–adrenal
IFN: Interferon
IL: Interleukin
IR: Ischemia–reperfusion
LPS: Lipopolysaccharide
MSC: Mesenchymal stem cell
MTx: Mitochondrial transplantation
NFκB: Nuclear factor kappa-light-chain-enhancer of activated B cells
OHCA: Out-of-hospital cardiac arrest
PCAS: Post-cardiac arrest syndrome
PD: Parkinson's disease
ROS: Reactive oxygen species
ROSC: Return of spontaneous circulation
TH: Therapeutic hypothermia
Treg: Regulatory T cells
TTM: Targeted temperature management
